# Life expectancy in multimorbid older adults: Why it matters for preventive care

**DOI:** 10.1093/eurpub/ckac129.291

**Published:** 2022-10-25

**Authors:** V Gastens, A Chiolero, D Anker, M Feller, DC Bauer, N Rodondi, C Del Giovane

**Affiliations:** 1 Institute of Primary Health Care, University of Bern, Bern, Switzerland; 2 Department of Community Health, University of Fribourg, Fribourg, Switzerland; 3 Department of General Internal Medicine, University of Bern, Bern, Switzerland; 4 School of Population and Global Health, McGill University, Montreal, Canada; 5 Departments of Medicine and Epidemiology, University of California, San Francisco, USA

## Abstract

Multimorbidity is highly prevalent among older adults and associated with a shorter life expectancy. Many guidelines recommend tailoring preventive care of multimorbid people according to life expectancy. Indeed, there is a time lag between a preventive care intervention and the expected potential benefit, and patients with a relatively short life expectancy might not have the time to benefit from the preventive care intervention. Further, both patients and health care providers tend to overestimate benefits and underestimate risks of interventions. It is therefore necessary to have a valid index for mortality prediction in multimorbid patients, but there is no life expectancy estimator designed and recommended for this population. The paper describes the development and internal validation of a new life expectancy estimator. In this presentation, we focus on the importance of life expectancy estimation in multimorbid older adults: Why does it matter in this population? What is the time lag to benefit of a preventive intervention, e.g., cancer screening? What is the state in this field, in research and clinical practice? How could tailoring preventive care to life expectancy improve patient outcomes?

